# A Case of Opium Poisoning Successfully Treated with Atropine and Caffeine

**Published:** 1885-07-20

**Authors:** Charles N. Dixon Jones

**Affiliations:** Brooklyn, Late House Surgeon to the Brooklyn Hospital


### A CASE OF OPIUM POISONING SUCCESSFULLY TREATED WITH ATROPINE AND CAFFEINE.

By CHARLES N. DIXON JONES, B.S., M.D., Brooklyn,
Late House Surgeon to the Brooklyn Hospital.

The following case is presented with the belief that it is of sufficient interest to be worthy of record :

Gertie E., aged eighteen months, was brought into the Brooklyn Hospital on the morning of Nov. 27, 1883, with all the symptoms of acute opium poisoning.

It was learned on inquiry that, two hours previously, the child had eaten six pills, each containing calomel two grains, opium one grain (the pills were chewed by an older brother and then given to his sister, who swallowed them.) Soon after, it was noticed that the child became very sleepy. A physician was sent for, who administered mustard in warm water ; slight vomiting followed, it was then given some strong coffee, which was retained on the stomach.

When admitted to the hospital, she was in a comatose condition, the pupils were insensible to light, and contracted to a pin-point in size. The respirations were frequent, shallow, and so feeble as to be barely perceptible. The pulse was small, weak and rapid. The face was cyanotic, extremities cold, and deeply congested.

Believing that the full emetic effect of the mustard had not been obtained, the indications to be met were: First, to overcome the immediate tendency to failure of respiration ; second, the evacuation of the stomach ; and, third, the administration of respiratory stimulants and the recognized antidote to opium.

A hypodermic injection containing 1.240 of a grain atropinæ sul. was accordingly given. As no immediate effect was produced. this was followed in ten minutes by another containing 1.120 of a grain, with the effect of increasing the force and frequency of the respirations, and slightly enlarging the pupil.

In the meantime I procured a stomach-pump, consisting of a flexible catheter (Nelaton's) twenty inches in length and one-third of an inch in diameter, connected, by means of a small piece of glass tubing, with a piece of rubber tubing three feet in length, and provided with a funnel.

The tube was introduced into the stomach with little difficulty. Lukewarm water was then slowly poured into the tube through the funnel. The washing was repeated three times before the fluid returned clear. The fluid removed from the stomach was found to contain a dark curd, consisting of the coffee which the child had swallowed, also the coatings, and several fragments of the pills which she had eaten. After each washing it was necessary to remove the tube and suspend the process, owing to the cyanotic condition of the patient.

The child was now placed in bed. The record taken at this time showed that the pulse was very rapid and feeble (with the stethoscope); the heart was beating at the rate of 160 a minute. The respirations were 6 per minute, superficial and gasping. In addition to the internal medication, external stimulation by means of hot spongiopilin applied over the præcordial region, hot-water cans to the extremities, and vigorous friction of the chest, legs and arms was kept up, and it was only after four hours unremitting care that reaction was established. Artificial respiration was practiced from time to time, when it was thought that life was in imminent danger from the failure or insufficiency of natural respiration.

At 2 P. M. her condition had somewhat improved. Hypodermic injection of atropine sulphate, gr. 1.120, was administered. A warm enema of olive-oil and soap-suds was given. No effect.

At 2.50 P. M., pulse 130, respiration 9.

At 3 P. M., citrate of caffeine, gr. j, spir. ammoniæ aromat. and brandy min. v, were administered.

At 3.30 P. M., caffeine repeated. Pulse 130, respiration 10.

At 4 P. M., caffeine repeated. Patient is startled when spoken to in a loud voice. A deadly pallor is noticeable about the alæ of the nose, the eyelids, and the margins of the auricles; other parts of the face congested. Later she showed a marked tendency to rub these parts. I am unable to explain the physiological phenomenon producing this appearance, but it is probably due to vaso-motor spasm, the drug having a selective affinity for the nerves supplying this particular region.

At 4.40 P. M., patient passed urine; small in amount and highly colored.

At 6 P. M., pulse 130, respiration 11.

At 7 P. M., caffeine, gr. ij.

At 8 P. M., hypodermic of atropine, grains 1.120.

At 9.50 P. M., respiration 14, pulse 140. From this time she rapidly improved until 12 M., when she began to show signs of returning life.

At 11.50 P. M., pulse 120, respiration 16. Bowels moved.

*28th.*—At 4 A. M., pulse 130, respiration 16.

At 6 A. M., pulse 120, respiration 18. The respirations are now regular for the first time; pulse is still rapid, but stronger. She now begins to show signs of returning consciousness.

During the day the child was very sleepy, but could be aroused to take nourishment. In the afternoon she was as bright and cheerful as if she had not passed through the terrible ordeal of opium poisoning. The after-treatment was the continuation of the milk diet, with a small amount of brandy, until evening, when she was discharged perfectly recovered.

It will be noticed in the history of this case that the first hypodermic injection of atropine was given at 11.45 A. M., the second at 11.55, and the third and fourth at intervals up to 8.10 P. M. The total quantity of sulphate of atropine given was about 1.35 of a grain. The quantity of citrate of caffeine used was five grains.—*New York Medical Journal.*

